# Out-of-hospital emergency medical services in Poland: organization, challenges, and modern solutions

**DOI:** 10.3389/fpubh.2026.1743569

**Published:** 2026-01-28

**Authors:** Krzysztof Marek Mitura, Daniel Celiński, Tadeusz Miłowski, Piotr Konrad Leszczyński, Jadwiga Snarska, Robert Gałązkowski, Sławomir Dariusz Szajda

**Affiliations:** 1 Independent Public Health Care Center RM-MEDITRANS Emergency Station and Sanitary Transport in Siedlce, Siedlce, Poland; 2 Department of Emergency Medical Service, Medical University of Warsaw, Warsaw, Poland; 3 Department of Emergency Medicine, Collegium Medicum, University of Warmia and Mazury in Olsztyn, Olsztyn, Poland; 4 Faculty of Medical and Health Sciences, University in Siedlce, Siedlce, Poland; 5 Department of Surgery, Collegium Medicum, University of Warmia and Mazury in Olsztyn, Olsztyn, Poland

**Keywords:** ambulance, emergency medical services team, emergency notification system, out-of-hospital emergency medical services, paramedic, Poland, pre-hospital emergency medical services, sudden health emergency

## Abstract

Emergency medical services systems are designed to provide medical aid in the event of sudden illness or injury. Each system comprises two parts, namely the out-of-hospital emergency medical services (OHEMS) and the in-hospital emergency medical services (IHEMS). The out-of-hospital component involves receiving emergency calls and providing medical services at the scene of an incident. The Polish OHEMS is mainly based on the Anglo-American model, with noticeable elements of the French-German model. Its fundamental principle is to aid any person who has found themselves in a state of sudden health emergency, which is one of the key responsibilities of the state. The organisational structure of the Polish system is multidimensional and linked to the administrative division of Poland. Its supervision, organisation and operation are the responsibility of the Minister of Health and regional governors. An emergency notification system has been established to handle emergency calls, where emergency medical dispatchers are responsible for responding to medical incidents. At the scene of an incident, medical aid is provided by emergency medical teams, including specialist, basic, motorcycle and airborne teams. Their personnel consist of system doctors, system nurses and paramedics. These teams operate within entities whose main shareholder is the State Treasury or a local government unit. A nationwide uniform ICT system supports the activities of emergency medical dispatchers and teams. The Polish OHEMS has numerous strengths, but is nevertheless subject to constant change due to the need to adapt to current needs and evolving conditions. At the same time, its role and effectiveness are highly valued by Polish society.

## 1 Introduction

Life and health are the most precious assets a person possesses. That is why states establish emergency medical services (EMS) systems, which provide rapid and effective aid in the event of sudden illness or injury.

The World Health Organisation (WHO) defines an emergency medical services system as the deployment of medical equipment and personnel that is capable of ensuring the effective operation and management of the system in the event of a hazard to human life or health (1). The WHO distinguishes between two basic components of the emergency medical services, namely out-of-hospital emergency medical services (OHEMS) and in-hospital emergency medical services (IHEMS) (1). The out-of-hospital component of the emergency medical services system refers to medical care provided at the scene of an incident, and begins when an emergency call is received by an emergency medical dispatcher, and ends when the emergency medical team (EMT) completes its activities at the scene of an incident, or after the patient has been transported to a healthcare facility. The task of the in-hospital component of the emergency medical services system is to provide medical services under hospital conditions to patients in a state of sudden health emergency, to be constantly on standby to perform them, and to continue medical activities initiated by the EMT.

Worldwide, most OHEMS are based on two main organisational models, namely the Franco-German and Anglo-American models (2, 3). The French-German model involves the presence of medical personnel on board ambulances and the provision of treatment to patients at the scene of an incident. In the case of the Anglo-American model, EMT personnel are typically trained paramedics, and patients are usually transported to emergency departments, rather than remaining at the scene of the emergency.

The Polish OHEMS combines elements of both models, yet in recent years it has been increasingly moving towards the Anglo-American model, which is due to the reduction in the number of teams including doctors, and the improvement in the qualifications and skills of paramedics, who are taking on more and more tasks in the area of patient care.

## 2 Methods

The study is a narrative review. Its primary objective was to describe the organisation and functioning of the Polish out-of-hospital emergency medical services, while identifying key challenges and modern solutions. To ensure a comprehensive overview of the Polish OHEMS, legislative, organisational, and clinical perspectives were integrated.

The review was based exclusively on published and publicly available materials. A non-systematic descriptive approach was used to summarise and interpret the literature relevant to the subject. A targeted literature search was conducted using electronic databases, including PubMed, Scopus, Google Scholar, and PPM.^1^ The search strategy employed a combination of English-language keywords: ‘out-of-hospital emergency medical services’, ‘OHEMS’, ‘prehospital emergency care’, ‘emergency medical team’, ‘EMT’, ‘emergency medical services’, ‘EMS’, ‘State Emergency Medical Services’, ‘ambulance services’, ‘Helicopter Emergency Medical Service’, ‘HEMS’, ‘paramedic’, as well as Polish-language terms: ‘Państwowe Ratownictwo Medyczne’ (State Emergency Medical Services), ‘zespół ratownictwa medycznego’ (emergency medical team), ‘ratownik medyczny’ (paramedic). The terms ‘Poland’ and ‘Polish’ were also included. Both English- and Polish-language publications were considered. Additional Polish-language sources were identified through national medical journals. Furthermore, the reference lists of selected articles were reviewed to identify additional relevant publications.

Publications were selected based on their relevance to the Polish OHEMS and included peer-reviewed articles, review papers, national reports, and legal acts regulating the emergency medical services system.^2^ The review covered literature published between 2015 and 2025. No strict inclusion or exclusion criteria were applied. In accordance with the assumptions of a narrative review, publications were not excluded on the basis of study design or potential risk of systematic bias. The literature analysis was qualitative, and the findings were interpreted in light of the authors’ clinical and academic experience.

The applied methodology is subject to potential selection bias and was not intended to provide an exhaustive or fully reproducible search of electronic databases. Nevertheless, it allows for a more in-depth understanding of the specific characteristics of the Polish OHEMS by integrating diverse sources that are sometimes underrepresented in systematic reviews. The authors also note the limited number of international and national peer-reviewed publications addressing the organisation and functioning of the Polish OHEMS.

## 3 Review

### 3.1 Historical overview

The first documented provision of medical care in emergency situations in Poland, as in other countries, was related to military operations (4). The first emergency medical station in Poland, and second in Europe, was established in 1891 in Kraków. Until the Second World War, a series of stations were established by municipal authorities, the Polish Red Cross, and social institutions (4, 5). These stations, however, had local coverage, mainly in large cities. After the end of the Second World War, efforts to establish emergency medical stations were resumed. Ultimately, it was as late as the 1950s that the national authorities addressed the legal regulation and organisation of the provision of medical aid in emergency situations, and, over time, the network of emergency medical stations covered the entire country (4, 5).

The establishment of Poland’s emergency medical services system began in the late 1990s. In 2001, the first Act on Emergency Medical Services was enacted, which defined the provision of medical aid to any person whose life or health was in danger as a task of the state (6). Later, on 8 September 2006, it was replaced by a new Act on State Emergency Medical Services (7). It remains in force today and, together with its implementing acts, regulates the organisation, operation and funding of the emergency medical services system, both in the out-of-hospital and in-hospital components. However, due to the challenges facing modern emergency medical services, it has been amended many times.

### 3.2 Basic guiding principles

**Figure 1:**
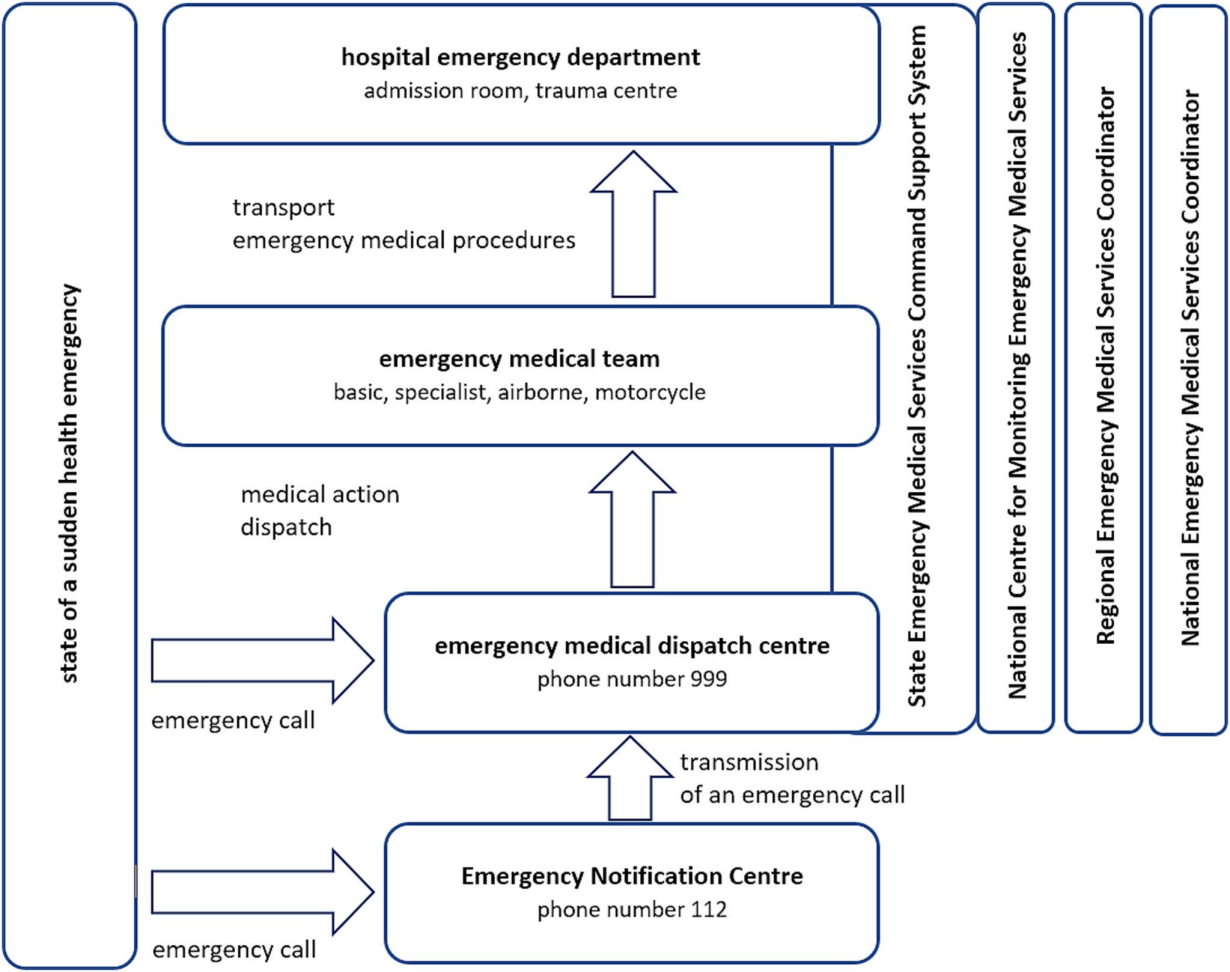
The organisational structure of the emergency medical services system in Poland.

The Act of 8 September 2006 also identified the provision of aid to any person in a state of sudden health emergency as a task of the state (7). The State Emergency Medical Service system, established under this Act, was designated to carry out this task. The State Emergency Medical Service system consists of OHEMS and IHEMS, i.e., emergency medical teams (EMTs) and hospital emergency departments, respectively (Figure 1). The Act defined a state of sudden health emergency as a state involving the sudden or anticipated appearance of symptoms of deteriorating health over a short period, which may result in serious damage to bodily functions, bodily injury, or loss of life. This state requires immediate emergency medical services and treatment. Emergency medical procedures should be understood as the provision of healthcare services, financed from public funds, provided by emergency medical teams. Both EMTs and hospital emergency departments provide healthcare services to people in a state of sudden health emergency, and are defined as system units.

It should also be stressed that, in addition to serving as a vital component of Poland’s broadly defined emergency medical services system, OHEMS also constitutes one of the elements of the state security system (8, 9).

### 3.3 Organisational principles

The structure of the Polish emergency medical services system is multidimensional, with the administrative division being of major importance for the organisation of the system (Figure 1). The State EMS system is supervised on a national level by the Minister of Health (7). The task of individual regional governors (Figure 1), representatives of the government administration in the field, is to supervise, plan, organise and coordinate the system in the regions under their authority (the highest territorial division level unit) (7).

As part of his/her duties, the Minister of Health may appoint a national emergency medical services coordinator (Figure 1), whose tasks include resolving disputes concerning the admission of patients in a state of sudden health emergency to hospital, and coordinating the cooperation between regional emergency medical services coordinators and emergency medical dispatchers from outside a single region (7). In turn, regional governors appoint regional emergency medical service coordinators (Figure 1), who also resolve disputes concerning the admission of patients in a state of sudden health emergency to hospitals, cooperate with emergency medical dispatchers, and coordinate their cooperation in the event of incidents requiring the use of EMTs from more than one operational area (7).

The State EMS system in each of the 16 regions operates on the basis of a regional action plan, drawn up by the regional governor with territorial competence (7). Each plan is subject to annual updates. Thus, each region has its own action plan, which must be approved by the Minister of Health as part of his/her supervisory role.

In their plans, regional governors indicate the operational areas of activity of the emergency medical dispatch centre. Furthermore, the plans include the number and location of EMTs and their areas of operation, determined on the basis of statutory parameters for arrival times at the scene of an incident, rules for cooperation between system units and cooperating units, and information on the location and number of emergency medical dispatchers, rules of operation, and the organisation of communication between EMTs, emergency medical dispatch centres and hospital emergency departments. The plans also provide data on the number and location of hospital emergency departments, as well as information on trauma centres for adults and children. The plans also include a range of statistical data, covering, for example, the response times of EMTs and the number of patients in hospital emergency departments. It can therefore be assumed that the organisation and functioning of the State EMS system in Poland is based on regional action plans.

When determining the number and distribution of EMTs, regional governors are obligated to ensure that an EMT arrives at the scene of an incident within the appropriate timeframe (7). These parameters are measured from the moment the call is received by the emergency medical dispatcher. These indicators include the median time to reach the scene of an incident, which should not exceed.

The organisation of the Polish OHEMS within a high-level administrative framework enables a high degree of organisational uniformity across the country. Local variations result primarily from geographical factors, the characteristics of the serviced area (urban versus rural), the road infrastructure, and the availability and profile of medical facilities within the operational area of individual emergency medical teams. A clearly defined division of responsibilities with regard to system supervision, control, planning, and development reduces competency-related disputes and allows the system to adapt relatively quickly to changing conditions. This was particularly evident during the COVID-19 pandemic, when the Polish OHEMS continued to function without significant disruption, while the main limitations were observed in in-hospital emergency medical services.

### 3.4 Emergency notification system

The emergency notification system (ENS) was established in Poland in 2013 (10). The ENS is founded on emergency notification centres (ENC), which are responsible for handling and forwarding emergency calls made to the emergency phone number 112 to the appropriate services. Emergency calls concern, among other things, the occurrence of sudden health emergencies, violence, safety hazards and threats to public order. The organisation, supervision and operation of the emergency notification system within the country is the responsibility of the Minister of the Interior and Administration. ENCs are established by individual regional governors, and their location and organisation are specified. Currently, there are 17 ENCs located in Poland, including a single ENC for the capital city of Warsaw. ENCs handle emergency calls, and then forward them to the appropriate police unit, fire brigade or emergency medical dispatch centre.

The reception and handling of emergency calls concerning sudden health emergencies, transmitted from the ENC, and received from the emergency phone number 999, is handled by emergency medical dispatch centres (Figure 1) (7). Before the introduction of the number 112, the emergency number 999 was used to report a sudden health emergency, and now coexists with the number 112. Until the end of 2020, emergency medical dispatch centres were subordinated to unit keepers, and since 2021, they have been organisational units of the relevant regional governors. Along with the establishment of the emergency medical services system in Poland, medical dispatch centres underwent changes, mainly in the form of a reduction in their number. In 2011, there were 337 of them (11), whereas there are currently 23 dispatch centres with 226 dispatch positions (12).

All emergency medical dispatch centres in Poland operate in a manner that ensures their replaceability in the event of a local ICT network failure or if all lines in a particular dispatch centre are busy. The tasks of emergency medical dispatch centres are carried out by emergency medical dispatchers, who may be persons with the qualifications required for system nurses or paramedics. The tasks of emergency medical dispatch centres involve receiving emergency calls, setting priorities, immediately dispatching EMTs to the scene of an incident, and instructing persons providing first aid. The work of emergency medical dispatchers is based on procedures that support decision-making regarding the acceptance or rejection of a call, the appropriate classification of the call, and the dispatch of EMTs to various states of sudden health emergency. Thus, emergency medical dispatch centres should receive calls and prioritise EMT departures based on an algorithm for collecting medical history (13). There are two priorities for EMT departures: code 1—immediate, and code 2—urgent. These determine the use of light and sound signals by the EMT, as well as the time within which the dispatcher must dispatch an EMT and the time within which the EMT must leave for an incident. EMT code 1 applies to incidents such as sudden cardiac arrest and loss of consciousness, while code 2 applies to, e.g., limb injuries and mental disorders.

Undoubtedly, the most important advantage of the Polish ENS is its nationwide uniformity. All emergency medical dispatchers operate according to uniform procedures for call handling and undergo mandatory, standardised training. Additionally, when call waiting times exceed acceptable limits, calls are automatically redirected to an available dispatcher, who may be located in another region but is still able to manage the incident and dispatch an emergency medical team.

### 3.5 Emergency medical teams

EMTs operate within healthcare entities, which the Act defines as unit keepers (7). At least 51% of such an entity must be owned by the State Treasury or a local government unit. The Act thus guaranteed that services provided under OHEMS are the domain of the state. Each of the ground-based EMT keepers concludes contracts for emergency medical services with the National Health Fund, the institution responsible for funding public healthcare through compulsory insurance contributions.

The specific nature of the activities of the EMT is based on remaining on a constant standby to perform emergency medical procedures under out-of-hospital conditions (14). Emergency medical teams must be equipped with specialised means of medical transport that meet the technical and quality standards required by law, and in the case of airborne teams, also those specified by aviation law. Each EMT’s vehicle must carry a minimum supply of medicines, equipment and medical supplies specified in an Order issued by the President of the National Health Fund (15). At the same time, the equipment and medicines available must be consistent with the qualifications of the paramedic, and in the case of specialist and airborne teams, they should be expanded in line with the needs and competencies of the doctors. In addition, every ambulance must comply with standard PN-EN 1789:2007 + A1 “Medical transport vehicles and their equipment. Road medical transport vehicles.” The standard specifies the minimum technical requirements for ambulances in terms of their design, safety, and equipment for transporting, treating, and monitoring patients. However, the equipment of ambulances largely depends on individual keepers. Some ambulances only carry the minimum equipment required by law, while others are equipped with critical parameter analysers, video laryngoscopes, or emergency ultrasound devices.

Polish EMTs are divided into specialist, basic, motorcycle and airborne teams (Figure 1). The main distinguishing feature of specialist EMTs is that they consist of at least three persons authorised to perform emergency medical procedures, including a system doctor and a system nurse or paramedic. A system doctor heads a specialist team. However, based on the experience gained during the COVID-19 pandemic, the legislator has allowed that during a state of epidemic hazard or a state of epidemic, a specialist team may consist of three persons qualified as paramedics or system nurses, with the team leader being one of these persons appointed by the unit keeper.

Regarding basic teams, there are two-person teams consisting of two authorised personnel, typically system nurses or paramedics, who are authorised to perform emergency medical procedures. There are also three-person teams consisting of three authorised personnel, typically system nurses or paramedics, who are authorised to perform emergency medical procedures. The head of the basic EMT is a person appointed by the unit keeper. In addition to the basic EMTs, seven water emergency medical teams operate during the holiday season to secure water bodies.

The specialist and basic teams may include a person without a medical educational background, who will act as a driver if none of the members of the EMT has a licence to drive an emergency vehicle. In addition, the legislator stipulated that for every 10 basic emergency medical teams, there should be at least one specialist team.

Motorcycle emergency medical services units were introduced in 2025 in response to demands from various circles. They comprise at least one person authorised to perform emergency medical procedures, who is a system doctor, system nurse or paramedic. There should be one emergency medical service motorcycle for every 400,000 inhabitants in a region, and it should operate from 1 May to 30 September, for a maximum of 12 h per day.

An airborne emergency medical team, on the other hand, typically consists of at least three personnel, including a professional pilot, a medical doctor, and a paramedic or nurse.

The division of EMTs into specialist teams (with a doctor) and basic teams (without a doctor) may create certain challenges in operational deployment. The absence of clear guidelines specifying which incidents should be handled by doctor-staffed teams and which by paramedic-staffed teams may pose organisational difficulties. In theory, specialist teams would be expected to manage incidents involving patients in potentially severe conditions, while basic teams would be dispatched to less serious cases. In practice, however, the unpredictability of call volumes and the actual clinical condition of patients encountered on scene renders such a dispatch model largely impractical. Consequently, the team able to reach the scene most rapidly is typically dispatched. It could be assumed that, within such a system, specialist teams should provide support to basic teams. In reality, however, basic EMTs only occasionally require assistance from specialist teams (16). This can be attributed to the high level of competence of paramedics and nurses working within the system, which largely compensates for the absence of a doctor in the ambulance. Moreover, available research indicates that the lack of a doctor on board an ambulance in Poland does not delay the initiation of treatment and does not adversely affect prognosis in patients with conditions such as acute coronary syndrome (17). At the same time, it should be emphasised that airborne EMTs provide substantial support to ground EMTs, particularly basic teams. Airborne EMTs units operate nationwide, are always staffed with a doctor, and enable rapid transport of patients to highly specialised medical facilities. Overall, it should be noted that there is insufficient evidence demonstrating clear benefits associated with the routine presence of doctors in EMTs (18, 19).

### 3.6 Medical personnel

Medical personnel forming part of the EMTs and airborne EMTs must have an appropriate educational background and experience. This applies to doctors, nurses and paramedics alike.

The initial assumption was that, ultimately, specialist teams and airborne EMTs should include a doctor specialising in emergency medicine, a field of medicine dealing with the diagnosis and treatment of patients in a state of sudden hazard to life or health. In Poland, this specialisation was established in 1999, but by 2023, only 1,132 doctors had obtained a specialisation in emergency medicine (20). Furthermore, in view of the fact that some of these doctors have not taken up employment in EMTs, doctors specialising in anaesthesiology and intensive care, general and paediatric surgery, internal medicine, cardiology, orthopaedics and traumatology, paediatrics and neurology, as well as doctors at various stages of acquiring the above specialisations, were also considered to be system doctors, i.e., doctors with the appropriate competences to work in EMTs.

In turn, the legislator has designated nurses with specialist titles, or those specialising in emergency nursing, anaesthesiology and intensive care, surgery, cardiology, paediatrics, as well as nurses who have completed a qualification course in the field of emergency nursing, anaesthesiology and intensive care, surgery, cardiology, paediatrics, as system nurses (7).

The largest professional group working in emergency medical teams are paramedics (21). Their training began in Poland in 1992. Initially, such qualifications could be obtained after completing post-secondary school, and since 2015, only by completing a degree in emergency medical services (22, 23). The training of paramedics as part of first-cycle studies began in 2000 (21), and since 2024, also as part of second-cycle studies (24). Obtaining professional qualifications is only the beginning of paramedic training. Paramedics are required to undergo continuous professional development, which must be documented and properly accounted for over five-year periods (25). For many years, the profession of paramedic has enjoyed high social prestige in Poland, translating into its ranking in first place among medical professions, and in second place, right after firefighters, among all professions (26).

Compared to other countries, both system nurses and paramedics have a very wide range of professional powers (9, 23). They can competently diagnose and provide initial treatment for states of sudden health emergency resulting from various illnesses and injuries (27, 28). Once the Act came into force, system nurses and paramedics could independently administer 26 medicines in predetermined forms and routes of administration (29). Currently, they can independently administer 50 medicines in various forms and by various routes. The range of emergency medical procedures they can perform includes simple wound dressing, fracture care, cardioversion, ultrasound scans, and certifying a patient’s death (30). For some time, there has also been debate concerning the possibility of administering muscle relaxants, which are necessary for performing Rapid Sequence Intubation (RSI).

### 3.7 State emergency medical services command support system

At the end of 2017, the State Emergency Medical Services Command Support System (SEMSCSS) was implemented throughout Poland (11). The SEMSCSS is a nationwide ICT system that enables the reception and recording of medical incidents, the localisation of emergency calls and EMTs, the dispatch of EMTs, and the generation of medical documentation by EMTs. The system helps medical dispatchers, EMTs, regional emergency medical service coordinators and the national emergency medical service coordinator perform their duties (7).

The Ministry of Health also uses the SEMSCSS for supervision and by regional governors for planning, organising, and coordinating the emergency medical services system. The SEMSCSS is continually being improved and refined, with new features being implemented to enhance the work of emergency medical dispatchers and EMTs.

The tasks of the Minister of Health include supervision of the SEMSCSS. The Minister is its administrator, determines the directions of development, and is responsible for the expansion and modification of the SEMSCSS. On behalf of the Minister of Health, the administration and responsibility for the expansion and modification of the SEMSCSS are vested in the National Centre for Monitoring Emergency Medical Services (NCMEMS), which operates within the structures of the Airborne Emergency Medical Team Keeper (7). The NCMEMS is also responsible for the proper functioning of the SEMSCSS and the 24/7 monitoring of incidents handled by emergency medical dispatchers and EMTs. In turn, individual regional governors ensure the maintenance and functioning of the SEMSCSS in their respective regions. In the current era of hazards posed by hybrid and terrorist attacks, such a centralised, uniform ICT system must be based on the country’s internal security systems, and must be highly resistant to various forms of destabilisation (31). To date, no serious hazards resulting from its destabilisation or cyber-attacks have been reported.

The data collected within the SEMSCSS derived from all EMTs interventions conducted in Poland, may serve as a valuable foundation for the implementation of quality and performance measures within the Polish OHEMS. Such measures could be based on time intervals, clinical data, patient and personnel data, or combinations of these variables. The SEMSCSS data also enable analyses and conclusions related to the epidemiology of diseases and injuries, trends in OHEMS utilisation, the effectiveness of primary health care, and the functioning of hospital emergency departments.

### 3.8 Course of medical action

**Figure 2:**
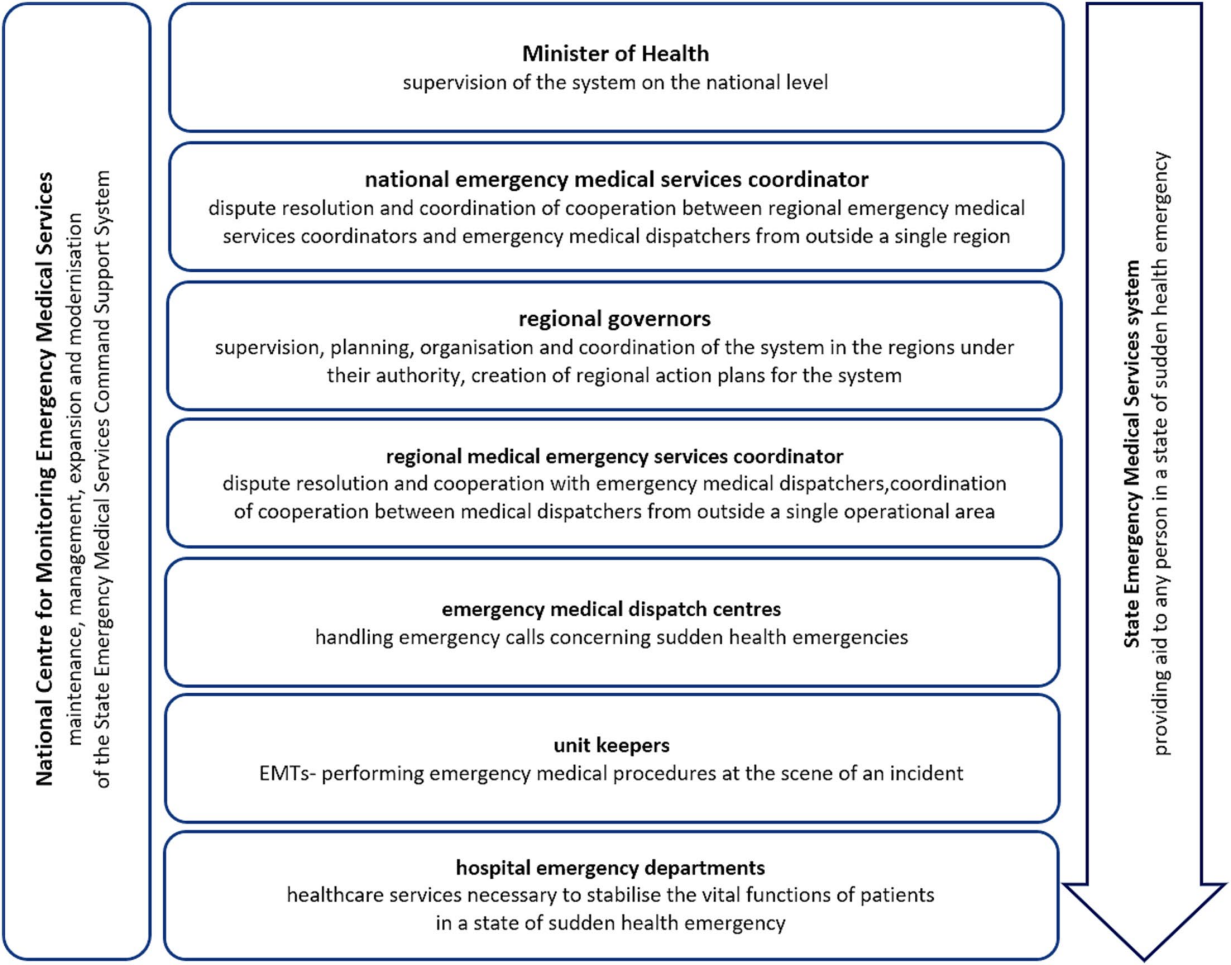
Course of medical action.

Under Poland’s legal conditions, aiding persons in a state of sudden health and life hazard may proceed in several stages (Table 1; Figure 2). Witnesses to an incident involving a sudden health emergency are already under a statutory obligation to immediately and effectively notify the entities responsible for providing aid to persons who have found themselves in such a state (7). Polish Criminal Code also imposes an obligation, under pain of criminal liability, to provide aid to persons in immediate danger of loss of life or serious damage to health (32). It can therefore be concluded that every adult person in Poland has a duty to provide first aid. Therefore, a witness to an incident involving a sudden health emergency should report the incident to the relevant services immediately. Calling the emergency number 112 will connect the witness to the ENC, where the operator will transfer the call to the emergency medical dispatcher after obtaining the necessary information. When calling 999, the witness will be directly connected to a medical dispatcher. After obtaining the necessary information, the emergency medical dispatcher will decide whether to accept or reject the call. If necessary, the dispatcher will then dispatch an EMT to the scene of an incident using the SEMSCSS and provide the caller with first aid instructions. As soon as the medical dispatcher receives the emergency call, medical action begins (Figure 2) (7).

**Table 1:** State of sudden health emergency, types of aid and entities responsible for providing it.

Type of aid	Entity responsible
First aid	Witness to an incident
Qualified first aid	Units cooperating with the system: fire protection units, the police, etc.
Emergency medical procedures	Emergency medical services teams: specialist basic, motorcycle, airborne
Provision of healthcare services	hospital emergency departments, trauma centres

If necessary, the emergency medical dispatcher, in cooperation with the relevant services, may dispatch units cooperating with the SEMSCSS system to the scene of an incident, whose paramedics, provided they have completed the appropriate training, provide so-called qualified first aid to persons in a state of sudden health hazard (Table 1; Figure 2) (7). Their actions are based on standard procedures and are mainly limited to providing basic care for the patient until an EMT arrives. The scope of activities that such a paramedic can perform includes, among others, manual and mechanical cardiopulmonary resuscitation, passive oxygen therapy, immobilisation of fractures, stopping bleeding, and dressing wounds. Such units include, e.g., fire protection units, the police, the Border Guard, and organisations whose statutory tasks include providing aid to persons in a state of sudden health emergency.

An emergency medical team arriving at the scene of an incident immediately begins performing emergency medical procedures (Table 1; Figure 2). After completing activities at the scene of an incident, the EMT will transport the person in a state of sudden health emergency to the nearest hospital emergency department, trauma centre or hospital indicated by the medical dispatcher, regional emergency medical services coordinator, or national emergency medical services coordinator (7).

Hospital emergency departments are also part of the State Emergency Medical Services system (Table 1; Figure 2). They are organisational units of hospitals and, at the same time, the primary destination to which EMTs transport patients. The hospital emergency department provides healthcare services, including initial diagnosis and treatment necessary to stabilise the vital functions of patients in a state of sudden health emergency (33). The hospital emergency department should be located to cover an area with a population of no more than 200,000 people, and an EMT should be able to reach it from the scene of an incident in 45 min or less.

In turn, trauma centres for adults and children, cooperating with the system, are intended for trauma patients who meet the criteria for anatomical injuries with concomitant physiological disorders (34). A single injury centre should serve a population of at least 1,000,000 people, and an EMT should be able to reach the centre within 1.5 h from the scene of an incident. It is also possible for an EMT to transport patients directly from the scene of an incident, bypassing the hospital emergency department, to specialised hospital units where medical services are provided, which significantly improves the prognosis for patients. Such organisational units of hospitals include, among others, haemodynamic laboratories and stroke units.

### 3.9 Funding

In Poland, emergency medical services provided by emergency medical teams, as well as medical aid in hospital emergency departments, are free of charge for every patient, regardless of whether they have insurance or the country from which they come.

The sources of system funding are specified in the Act on State Emergency Medical Services (7). The operation of emergency medical dispatch centres is funded from the state budget, from the portions allocated to individual regional governors. The funding of EMTs is the responsibility of the National Health Fund, which allocates public funds for healthcare services. Thus, the National Health Fund combines tasks related to planning, spending and accounting for funds allocated for the operation of EMTs, ensuring their proper functioning. This method of funding EMTs may raise some concerns, as the financial plan limits the National Health Fund’s decisions on the purchase of services, and the National Health Fund cannot make commitments greater than those included in the plan (35). In turn, the funding of airborne EMTs comes from the state budget, from the portion allocated to the Minister of Health. Tasks essential to the functioning of the SEMSCSS system are funded from the state budget, from both the part administered by the Minister of Health and from the parts administered by the relevant regional governors. However, healthcare services provided by hospital emergency departments are funded from public funds, within the limits specified in the National Health Fund’s financial plan.

### 3.10 Functioning of the system

Poland covers an area of 313,933 km^2^ and, at the end of 2024, it had a population of 37,489,087 (36). Throughout 2024, emergency medical dispatchers handled 5,083,448 calls, of which 1,794,664 (35.3%) were refused the dispatch of an EMT (12). At the same time, there were 1,664 ground-based EMTs operating in Poland, comprising 248 (14.9%) specialist teams and 1,416 (85.1%) basic teams, as well as 21 airborne EMTs (37, 38). These teams provided medical aid to a total of 3,153,687 people. In 2015, there were 1,486 EMTs, including 588 (39.6%) specialist teams, 898 (60.4%) basic teams, and 17 airborne EMTs, which together provided aid to 3,168,451 patients (37). In 2024, the index of the number of EMTs per 100,000 inhabitants across Poland was 4.4, while in individual regions, it ranged from 3.8 to 6.2. In contrast, in 2015, the index for the entire country was 3.9, ranging from 3.3 to 5.5 across different regions. It is therefore evident that, over a 10-year period (Table 2), with a similar number of EMT interventions, there has been an increase in the overall number and density of EMTs, accompanied by a pronounced decrease in the proportion of specialist teams to basic teams.

**Table 2:** Number of emergency medical teams, their types and interventions in 2015 and 2024.

Emergency medical teams	Year	
	2015	2024
Total	1,486 (100.0%)	1,664 (100.0%)
Including: specialist teams	588 (39.6%)	248 (14.9%)
Basic teams	898 (60.4%)	1,416 (85.1%)
Airborne EMTs	17	21
Total number of interventions	3,168,451	3,153,687

The observed change in the ratio of doctor-staffed to non-doctor-staffed EMTs may be attributed to three main factors: difficulties in recruiting and retaining doctors, the high professional qualifications of paramedics and nurses within the system, and the higher costs associated with maintaining specialist emergency medical teams. The increase in the overall number of EMTs over the past decade can, in turn, be explained by the need to ensure timely and effective medical assistance in response not only to growing public demand, but also to the frequent exceeding of statutory response time limits by existing teams.

In 2024, the index of EMT interventions per 1,000 inhabitants was 84, ranging from 61 to 101 depending on the region (38). In 2024, EMT interventions for women (50.1%) outnumbered those for men (49.9%). In turn, the largest age group of patients who received aid from EMTs was those aged 65 and over (51.4%), followed by those aged 18 to 64 (42.6%) and those under 18 (6.0%). In 1.7% of cases, EMTs did not take any action due to the patient’s death. The most common call-out destination was the patient’s home (76.7%).

At the end of 2024, 20,255 people were employed in EMTs, with an EMT being the main place of work for only 12,900 employees (38). The largest group comprised paramedics (11,400), followed by system nurses (over 1,000), system doctors (over 200) and other personnel (drivers, pilots) who accounted for 300 people.

The time specified in the Act for an EMT to reach the scene of an incident, calculated from the moment the emergency medical dispatcher receives the call to the arrival of the EMT at the scene, seems to be one of the basic indicators used to assess the effectiveness and functioning of the SEMSCSS system. Data for 2023 shows that the median time (a maximum of 8 min) for an EMT to reach the scene of an incident in cities with more than 10,000 inhabitants was exceeded in all regions, and in areas outside cities with more than 10,000 inhabitants (a maximum of 15 min), it was not exceeded in only 1 out of 16 regions (39).

Starting from the beginning of the COVID-19 pandemic, there has also been a nearly threefold increase in the percentage of EMT interventions that were not related to a sudden health emergency. In 2019, the percentage of such departures was 20.9% of all departures, whereas in 2023, it increased to 58.7% and, depending on the region, ranged from 47.7 to 69.2% (39).

### 3.11 Strengths and weaknesses of the Polish emergency medical services system

The Polish OHEMS has numerous advantages, but the problems it faces necessitate constant changes to its organisation and adaptation to current needs. The greatest advantage of the Polish emergency medical services system is that the services it provides are the responsibility of the state, and providing aid in the event of a sudden health emergency is free of charge, regardless of the patient’s country of origin, financial situation, or insurance status.

Another advantage is that the system is uniform throughout the country, with standardised procedures for calling for aid, receiving calls, and operating EMTs. A further advantage is the well-trained and highly competent ambulance personnel. The SEMSCSS, a nationwide ICT system for handling incidents related to sudden health emergencies, also appears to be a strong point of the Polish system. It should be noted, however, that the SEMSCSS, as a centralised system, must be very stable and resistant to various forms of destabilisation.

The division of EMTs into specialist and basic, two- and three-person teams may present difficulties in their dispatch. This is due to the lack of regulations specifying which medical incidents require a specialist team to be dispatched and which require a basic team. Having specialist EMTs stationed mainly in cities and basic EMTs in rural areas may result in accusations of unequal access to medical services. All this is exacerbated by personnel shortages among medical personnel, particularly doctors specialising in emergency medicine, and an insufficient number of doctors with this specialisation. Therefore, the system should aim to rely solely on basic teams, providing them with medical support using new technologies, sending a doctor to the ambulance if necessary (rendezvous system), or extending the competencies of paramedics and system nurses to include RSI. The rendezvous system may prove effective in large cities, but in rural areas, its main limitation may be the time it takes for the doctor to reach the EMT.

A significant burden on the system, which can also result in delays in obtaining aid by patients in life- and health-threatening situations, is the large number of EMT departures that do not meet the criteria for a sudden health emergency.

Despite standards regarding the minimum equipment for EMTs, the equipment on ambulances varies depending on the ambulance’s keeper. For some of them, emergency medical services are not their primary form of medical activity; often, it is hospital treatment. Thus, there is a clear fragmentation among keepers, who may be responsible for operating several to several dozen ambulances in the system, i.e., for a small area of a region or even for the entire region.

The funding of EMTs may also raise certain concerns due to restrictions on the spending of public funds as part of providing healthcare services, which results in a lack of financial flexibility during the fiscal year.

The weak point of the Polish OHEMS is the lack of quality indicators beyond those specified in the Act, such as the time taken by an EMT to reach the scene of an incident, as well as the median and maximum times taken to reach the scene. Statutory response times for EMTs to reach the scene of an incident have been exceeded numerous times for years, both in urban and rural areas, and increasing the number of EMTs is insufficient to compensate for this. Therefore, other indicators should also be developed, primarily based on the performance of emergency medical procedures and supported by scientific research. Indicators relating to the time it takes to reach the scene should only cover interventions involving an immediate hazard to life and health.

Measures typically provide quantitative results that can be used as benchmarks or guidelines for improving the quality and effectiveness of medical services (40). Although the development of universal measures for OHEMS may appear challenging due to the wide range of scenarios and task combinations encountered by emergency medical teams, it remains feasible. Currently, numerous quality measures of pre-hospital care are applied worldwide. Most of these are based either on time intervals or on large cohorts of patients with specific conditions (41, 42), or injuries (43), and the Delphi methodology is frequently employed in their development (42, 44).

A variety of quality measures could be readily implemented within the Polish OHEMS using data from the SEMSCSS, derived from the handling of emergency incidents by medical dispatchers and EMTs. These data enable not only the assessment of call handling based on time intervals, but also the evaluation of service quality using clinical data and the analysis of overall system efficiency. Measures developed on the basis of SEMSCSS data should additionally be complemented by measures reflecting the satisfaction of key stakeholders, including patients and EMS personnel.

However, as noted previously, apart from the verification of response times for individual stages of call handling, the Polish OHEMS has not yet implemented other quality measures that are commonly used internationally. Nevertheless, a number of scientific studies address issues related to the quality of pre-hospital care in Poland and the implementation of quality measures based on clinical data, including those related to sudden cardiac arrest (45), pain management (46, 47), and acute coronary syndromes (17). Other studies focus on system efficiency, particularly in the context of the COVID-19 pandemic (48), as well as on trends in the utilisation of emergency medical services (28).

### 3.12 Polish OHEMS compared to other European Union countries and the United Kingdom

The primary objective of the European Union (EU) is cooperation among Member States, aimed at strengthening solidarity and improving economic, social, and territorial cohesion. Despite this overarching goal, significant differences persist in the organisation of OHEMS between individual countries and even between regions within the same country.

In Poland, out-of-hospital emergency medical services are a state responsibility, and the entire system is centrally organised and largely uniform nationwide. Similar system uniformity can be observed, for example, in the Czech Republic, Slovakia (49), and France (50). In contrast, marked regional variation in OHEMS organisation within a single country is characteristic of Germany (18), Sweden (51), Italy (9), and Spain (52). In the United Kingdom, emergency medical services are provided by 14 regional organisations that operate as integral components of the public health care system (50).

Considering the division of EMTs into specialist (doctor-staffed) and basic (non-doctor-staffed) teams, the Polish OHEMS can be classified as a mixed or hybrid system. It is predominantly based on the Anglo-American model, while incorporating selected elements of the French-German model. This hybrid approach currently represents the most common organisational solution among European countries. The French-German model is applied, for example, in Austria, Belgium, Croatia, France, Germany, Italy, and Slovakia (18). Systems characterised by limited doctor involvement predominate in Scandinavian countries, Iceland, and the United Kingdom (18). In Northern European countries such as Sweden, the Netherlands, Iceland, Finland, and Norway, doctor involvement is largely restricted to helicopter emergency medical services (18). Ireland and Cyprus are the only European countries in which doctors do not participate in emergency medical services at all (18). Additionally, in 17 EU countries, primary care doctors participate in the management of sudden health emergencies after being notified by coordination centres (18).

Polish paramedics, similarly to their counterparts in England, Finland, Germany (Notfallsanitäter), and Norway, are required to hold at least a bachelor’s degree (9). In contrast, university-level education is not mandatory for paramedics in Denmark, Iceland, France, and Italy (9). At the same time, Czech paramedics have a narrower scope of practice than their Polish, Slovak, and German counterparts, with certain procedures permitted only under direct medical orders (49).

In the Czech Republic, EMTs may consist of a doctor and a paramedic, or be composed exclusively of paramedics (49). To support non-doctor-staffed teams, a rendezvous system is employed, whereby paramedics arrive first at the scene and a doctor follows after receiving a report from the team. A similar organisational model exists in Slovakia, where two types of EMTs operate: doctor-staffed teams and teams consisting solely of paramedics (53).

In Germany, OHEMS are organised in a highly heterogeneous manner, and the performance of invasive procedures on critically ill patients is regulated locally by medical directors (54). The system is based on paramedic-staffed EMTs, with on-scene support provided by doctors arriving via a rendezvous system (55). Thus in the German system a doctor plays a main role.

French OHEMS operate through teams composed of doctors, nurses, and paramedics (50). In addition, France maintains fire brigade–based ambulance services providing basic medical care. Due to the specific structure of the system, the role of paramedics is largely auxiliary.

The Italian OHEMS comprises three types of teams: basic teams staffed by two paramedics; intermediate teams consisting of paramedics and nurses; and advanced teams composed of a doctor, a nurse, and at least one paramedic (9). Teams without doctors are authorised to perform basic life-saving procedures, while medical interventions and drug administration are carried out in accordance with established protocols. Paramedic training in Italy is not standardised.

In the United Kingdom, EMTs personnel include paramedics and emergency medical technicians (50). English paramedics are highly qualified and have extensive professional competences. The efficiency and quality of services provided by each OHEMS organisation are systematically monitored and evaluated by public authorities, based on mandatory audits, collected clinical data, patient satisfaction assessments, response time measures, etc.

In Sweden, ambulance crews typically consist of one nurse and one paramedic. Nurses are qualified to perform advanced resuscitation procedures, whereas paramedics are responsible for basic life-saving interventions (51).

The Spanish emergency care model varies considerably between regions and is closely integrated with primary health care. In Asturias, integration between primary care and OHEMS is based on standardised protocols and applies to both urban and rural areas (52). In urban settings, after a doctor or nurse receives an emergency call, either an advanced team - including a doctor if required - or a basic paramedic-staffed team is dispatched. In parallel, a nearby primary care emergency team may also be mobilised. In rural areas, a primary care doctor is often the first healthcare professional to reach the patient and subsequently determines the need for dispatching an emergency medical team and its composition.

In summary, OHEMS differ substantially across European countries. While many systems display characteristics of the Anglo-American model, the French-German model, or a combination of both, each country has developed its own organisational solutions shaped by specific systemic conditions. Poland has likewise developed a distinct OHEMS model influenced by multiple systemic factors. Experience from recent years demonstrates that the Polish system is characterised by considerable flexibility and a high capacity for adaptation, as evidenced by its rapid response to emerging challenges.

## 4 Limitations

This study has several limitations. These stem from its reliance on national administrative and registry data, the absence of analyses of patient-level treatment outcomes, the lack of detailed consideration of regional differences within Poland, and the limited inclusion of quality dimensions such as personnel workload, burnout, and patient experiences. Additionally, the number of international and national peer-reviewed publications addressing the organisation of the Polish OHEMS is limited, and some available studies are partially or fully outdated.

## 5 Summary

One of the tasks of the Polish state is to provide aid to any person who finds themselves in a sudden state of health emergency. The performance of these tasks is largely the responsibility of OHEMS, which is also an element of state security.

The organisational structure of the Polish system is closely tied to the country’s administrative division. Its supervision is the responsibility of the Minister of Health and regional governors. The organisation of the system relies on regional action plans developed by individual regional governors.

Compared with other European countries, the organisation of the Polish OHEMS exhibits several distinctive features. The system is organisationally uniform nationwide, the equipment of emergency medical teams is standardised to a significant extent, and a unified information and communication technology system supports both medical dispatchers and EMTs in the management of emergencies. The scope of practice of paramedics is broad and consistent across the country. At the same time, the increasing implementation of modern, technology-based solutions is evident. The transmission of electrocardiogram recordings is standard practice, and the transmission of ultrasound images to consulting doctors or real-time streaming of cardiac monitor data is becoming increasingly common.

Emergency calls concerning medical incidents are handled directly by emergency medical dispatchers, who perform their duties in accordance with procedures and using an ICT system. Appropriate medical aid at the scene of an incident is provided by a network of EMTs, which are divided into specialist, basic, motorcycle and airborne teams, depending on their personnel composition and the specialist means of transport used. These teams are on a constant standby to provide immediate medical aid under emergency medical conditions at the scene of an incident, followed by the transport of patients to emergency departments. At the same time, Polish EMT personnel, including system doctors, system nurses, and paramedics, possess the appropriate educational background and extensive skills to diagnose and provide appropriate medical care at the scene of an incident. It should therefore be pointed out that proper organisation of the system enables saving lives at the scene of an incident, transporting and treating patients at the appropriate medical facility in accordance with medical indications.

Despite its many positive aspects, the Polish OHEMS requires further organisational measures to address current needs, changing conditions, and technological developments. The development of clear standards and even more effective organisation will translate into the effectiveness of activities, and an improvement in the quality of medical services provided. It may also be important to introduce quality indicators beyond those related to the statutory times for an EMT to reach the scene of an incident. Enhancing quality must be based on three main pillars: properly trained medical personnel, modern equipment, and continuous supervision of the medical services provided.

## Footnotes

1 https://ppm.edu.pl

2 https://isap.sejm.gov.pl
